# Obeticholic acid reduces biliary and hepatic matrix metalloproteinases activity in rat hepatic ischemia/reperfusion injury

**DOI:** 10.1371/journal.pone.0238543

**Published:** 2020-09-10

**Authors:** Andrea Ferrigno, Giuseppina Palladini, Laura Giuseppina Di Pasqua, Clarissa Berardo, Plinio Richelmi, Massimiliano Cadamuro, Luca Fabris, Stefano Perlini, Luciano Adorini, Mariapia Vairetti

**Affiliations:** 1 Dept. of Internal Medicine and Therapeutics, University of Pavia, Pavia, Italy; 2 Fondazione IRCCS Policlinico San Matteo, Pavia, Italy; 3 Dept. of Molecular Medicine (DMM), University of Padua, Padua, Italy; 4 Department of Internal Medicine, Liver Center and Section of Digestive Diseases, Yale University, New Haven, CT, United States of America; 5 Emergency Department, Fondazione IRCCS Policlinico San Matteo, Pavia, Italy; 6 Intecept Pharmaceuticals, San Diego, CA, United States of America; Texas A&M University, UNITED STATES

## Abstract

**Background:**

We have previously shown that obeticholic acid (OCA) upregulates the biliary excretion of asymmetric dimethylarginine (ADMA), an inhibitor of iNOS regulating the activity of matrix metalloproteinases (MMPs). Here, the effects of OCA on MMP-2 and MMP-9 activity in liver, bile and serum were evaluated after hepatic ischemia/reperfusion (I/R) injury.

**Material and methods:**

Male Wistar rats (n = 20) were orally administered 10 mg/kg/day of OCA (5 days) and subjected to a 60-min ischemia and 60-min reperfusion. Bile, serum and tissue were collected for MMP-2 and MMP-9 activity quantification. The MMP regulator tissue reversion-inducing cysteine rich protein with Kazal motifs (RECK), tissue inhibitor of metalloproteinases (TIMPs), iNOS and biliary levels of LDH, γGT, glucose and ADMA were quantified.

**Results:**

In the I/R group, OCA administration reduced MMP-2 and MMP-9 in liver, bile and serum. A downregulation of tissue RECK and TIMPs, observed under I/R, were recovered by OCA. Immunohistochemical staining of hepatic tissue demonstrated that RECK expression is mainly localized in both cholangiocytes and hepatocytes. Hepatic iNOS positively correlated with tissue MMP-2 and MMP-9 activity. Biliary levels of LDH, γGT and glucose were lower in I/R rats treated with OCA; in bile, MMP levels positively correlated with LDH and γGT.

**Conclusion:**

Thus, OCA administration confers protection to cholangiocytes via downregulation of biliary MMPs in livers submitted to I/R. This event is associated with hepatic RECK- and TIMP-mediated MMP decrease.

## Introduction

Obeticholic acid (OCA), a bile acid derivative and potent farnesoid X receptor (FXR) agonist, has been approved in the US and Europe for the treatment of primary biliary cholangitis (PBC) [1–3]. In addition, a multicentre, randomized, placebo-controlled trial (FLINT) evaluating the efficacy of OCA in patients with non-alcoholic steatohepatitis (NASH), has reported a decrease in the non-alcoholic fatty liver disease (NAFLD) fibrosis score in the OCA group (45%) versus the placebo group (21%). OCA was associated with improvement in hepatic steatosis, inflammation, ballooning and fibrosis [4, 5].

We recently documented, in a model of rat ischemia/reperfusion (I/R) injury, the ability of OCA to upregulate the biliary excretion of asymmetric dimethylarginine (ADMA), a potent inhibitor of constitutive and inducible nitric oxide synthase (NOS) [6]. We also demonstrated OCA’s ability to decrease iNOS content. This enzyme is involved in the pathogenesis of hepatic I/R injury as it synthesizes NO causing hemodynamic instability [7, 8].

Recently, the reversion-inducing cysteine-rich protein with Kazal motifs (RECK), a protease inhibitor-like molecule anchored to the plasma membrane, was identified as a novel transcriptional target gene of the FXR [9].^.^ RECK, which can be detected in a wide variety of normal human tissues, functions as a modulator of matrix metalloproteinases (MMPs) [10]. Moreover, an FXR agonist, WAY-362450, has been shown to augment hepatic RECK protein expression in mice fed a methionine and choline deficient diet [9].

Historically, MMPs were thought to function mainly as enzymes degrading structural components of the extracellular matrix (ECM). However, in the last few years, MMPs have assumed the important role of mediators of physical adaptation in tissues, whether developmentally regulated, environmentally induced or disease associated [11]. A new and more complex understanding of the role of MMPs during I/R is emerging. The action of MMPs can go beyond the ECM destruction or the recruitment of leukocytes in the hepatic parenchyma [12]. Although tissue inhibitor of metalloproteinases (TIMPs) are considered the principal endogenous inhibitors of MMPs, the regulation of MMP activity is a complex and multifaceted process; in particular, the relationship between iNOS-dependent nitric oxide (NO) and MMP-9 induction has received much attention over the last few years [13]. Hamada et al. showed that specific iNOS inhibition significantly down-regulates MMP-9 activity and disrupts leukocyte migration in hepatic I/R injury [14]. The authors also highlighted that the iNOS-derived NO regulated neutrophil transmigration occurred across fibronectin in an MMP-9-dependent manner.

In the present study, we undertook investigations into the effects of OCA on hepatic, biliary and serum levels of Gelatinases, MMP-2 and MMP-9, during I/R injury. In addition, we investigated whether OCA treatment modulates hepatic RECK and iNOS content as well as modulating cholangiocyte function and injury.

## Materials and methods

### Materials

All reagents were of the highest grade of purity available and were obtained from SIGMA (Italy). The FXR agonist, OCA, was kindly provided by Intercept Pharmaceuticals, San Diego, California, USA.

### Animals and experimental design

Male Wistar rats (Harlan-Nossan, Italy) were used in this study. The animals (n = 24) were allowed free access to water and food in all the experiments. The use and care of animals in this experimental study was approved by the Italian Ministry of Health and by the University of Pavia Commission for Animal Care (Document number 179/2017-PR). Animals were orally administered 10 mg/kg/day of the OCA in methylcellulose 1% vehicle for 5 days (n = 12) or with vehicle alone (n = 12). The effects of I/R were studied *in vivo* in a partial normothermic hepatic I/R model (I/R n = 6 and I/R + OCA n = 6). The rats were anesthetized with sodium pentobarbital (40 mg/kg i.p.), the abdomen was opened via a midline incision and the bile duct was cannulated (PE-50) [15]. Ischemia to the left and the median lobe was induced for 60 min with microvascular clips by clamping the branch of portal vein and the branch of the hepatic artery after the bifurcation to the right lobe, with the abdomen temporarily closed with a suture [16]. After 60 min of ischemia, the abdomen was reopened, the clips were removed, the abdomen was closed again, and the liver was allowed to reperfuse for 60 min. By using partial, rather than total, hepatic ischemia, portal vein congestion and subsequent bacterial translocation into the portal venous blood was avoided. Sham-operated animals were subjected to the same procedure without clamping the vessels (Sham-operated n = 6 and Sham-operated + OCA n = 6). To prevent postsurgical dehydration and hypotension, 1 ml of saline was injected into the inferior vena cava. All the animals were maintained on a warm support to prevent heat loss with rectal temperature at 37±0.1°C. Animals were sacrificed, under general anesthesia (sodium pentobarbital 40 mg/kg i.p.), by exsanguination.

### Serum, bile and tissue sampling

Blood was drawn from the vena cava and allowed to clot at room temperature. Serum was centrifuged at 3000 g for 10 min at 4°C. Bile samples were collected in darkened tubes. Hepatic biopsies were quickly removed from the median lobe and immediately frozen in liquid nitrogen, as were bile and serum samples.

### Biochemical assays

Liver injury was assessed by serum levels of alanine transaminase (ALT), aspartate transaminase (AST) and alkaline phosphatase (ALP) using standard commercial kits (Merck, Italy). Biliary γGT and glucose were evaluated by standard commercial kits (Merck, Italy). Biliary LDH was evaluated as previously reported [17]. ADMA levels in plasma, tissue and bile were evaluated by direct ELISA kit according to the manufacturing procedure (Immundiagnostik AG-Germany) [18, 19].

### Gelatin zymography

After sacrifice, median hepatic lobes were quickly excised and placed in cold (4°C) buffer (30 mM Histidine, 250 mM sucrose, 2 mM EDTA, pH 7.2) to remove blood. Liver was weighed and subsequently cut, frozen in liquid nitrogen, and stored at −80°C until use. Hepatic MMPs were extracted by homogenization (IKA-ULTRA TURRAX T10) of frozen liver tissue. Protein extraction from snap-frozen liver tissue, bile and serum and gelatin zymography were performed as described previously [20]. Briefly, the homogenate sample protein content was normalized by a final concentration of 400 μg/mL in the sample loading buffer (0.25 M Tris-HCl, 4% sucrose w/v, 10% SDS w/v, and 0.1% bromophenol blue w/v, pH 6.8). After dilution the samples were loaded onto electrophoretic gels (SDS-PAGE), containing gelatin, under nonreducing conditions followed by zymography. After electrophoresis and incubation, gels were stained with Coomassie Blue. Quantification of proteinases activity, by densitometer analysis, (GS 900 Densitometer BIORAD, Hercules, CA, USA) was expressed as optical density (OD), reported to 1 mg/mL protein content.

### Western Blot assay

CelLytic Buffer and Protease Inhibitor Cocktail were purchased from Sigma-Aldrich (Milan, Italy), as well as the mAb anti-alpha-tubulin (DM1A). Rabbit polyclonal antibody directed against iNOS was purchased from Cayman Chemical (Ann Arbor, Michigan, USA). Mouse monoclonal antibody against RECK was purchased from Santa Cruz Biotechnology, as well as rabbit polyclonal antibody against actin. Mouse monoclonal antibodies against TIMP-1 and TIMP-2 were purchased from Thermo Fisher Scientific (USA). Liver tissue samples were homogenized in an ice-cold CelLytic Buffer supplemented with Protease Inhibitor Cocktail and centrifuged at 15000 g for 10 min. The collected supernatant was divided into aliquots containing the same amount of proteins and stocked at -80°C. Samples of liver extracts containing the same amount of proteins were separated in SDS-PAGE on 7.5% or 12% acrylamide gels and transferred to PVDF membrane. Unspecific sites were blocked for 2 hours with 5% Bovine Serum Albumin (BSA) in TBS (20 mM Tris/HCl, 500 mM NaCl, pH 7.5, 0.1% Tween 20) at 4°C. The membranes were incubated with primary antibodies overnight at 4°C, under gentle agitation. Primary antibodies against alpha-tubulin, RECK and iNOS were used at a dilution of 1:1000. Anti-TIMP-1 and TIMP-2 was used at 1:200. Anti-actin was used at the dilution of 1:5000. Membranes were washed in PBS (Na_2_HPO_4_ 8 mM, NaH_2_PO_4_-H_2_O 2 mM, NaCl 140 mM, pH 7.4, 0.1% Tween 20) and incubated with peroxidase-conjugated secondary antibody at a 1:1000 dilution for RECK, TIMP-1, TIMP-2 and actin; at a 1:2000 dilution for alpha-tubulin and iNOS. Immunostaining was revealed with BIO-RAD Chemidoc XRS^+^. Bands intensity quantification was performed by BIO-RAD Image Lab software.

### Immunohistochemistry, immunofluorence and histological evaluation of biliary structures, hepatic progenitor cells and RECK

Acetone-fixed, 4 μm thick, cut frozen tissue section from rats following ischemia reperfusion (I/R n = 6) and ischemia reperfusion + OCA treatment (I/R + OCA, n = 6) were immunostained with anti-Wide Spectrum Screening Cytokeratins antibody (pCK) (Rabbit polyclonal, 1:400, Agilent), phospho-p42/44 (Rabbit monoclonal, p-p42/44) (1:400, Cell Signaling) and cleaved caspase 3 (CC3) (Rabbit polyclonal, 1:200, Cell Signaling). Following 1 h incubation at room temperature with primary antibodies, slides were rinsed in PBS 1x (Sigma Aldrich) and then incubated for 30 min at room temperature with the appropriate secondary antibody (EnVision, DAKO). Sections were then rinsed in PBS 1x and developed with 3,3'-Diaminobenzidine (DAB, Sigma-Aldrich) peroxidase substrate, counterstained with Gill’s n°2 hematoxylin, dehydrated in Ethanol and Xylene (both from Carlo Erba), and finally mounted using Eukitt® (Bio-Optica). For immunofluorescence, following re-hydration of the slides, samples were incubated overnight at 4°C with RECK (Mouse monoclonal, 1:100, Santa Cruz) and pCK (Mouse monoclonal, 1:100 BD Biosciences) (as marker of biliary structures). Following incubation, slides were rinsed with PBS 1x and then incubated 45mins with the appropriate secondary antibody (Alexa fluor 488 or 594, 1:500, ThermoFisher). Slides were then mounted with Vectashield + DAPI (Vector). In both immunohistochemistry and immunofluorescence, aspecific staining were blunted pretreating samples for 10mins with Protein Block (Leica). Immunostainings and immunofluorescences were analyzed with a Nikon Eclipse E800 microscope equipped with a DS-U1 cooled digital camera (Nikon) and analyzed with NIS software (Nikon). Morphometric analysis was used to quantify biliary structures and hepatic progenitor cells (HPC) according with the categorization of Roskams et al. [21]. Biliary structures were defined as pCK positive structures with biliary phenotype arranged in irregularly shaped structures inside the portal area or at the portal interface, whilst HPCs were recognized as pCK positive small cells with oval, or spindle-shaped morphology, alone or in small clamps, presents within the parenchyma or at the portal interface. Number of biliary structures and HPCs were counted in 5 non-overlapping randomly-taken fields taken at 200x and for each picture, number of structures were normalized on the number of portal tracts.

### Statistical analysis

Statistical Analysis was performed using MedCalc Statistical Software version 18.11.3 (MedCalc Software bvba, Ostend, Belgium; https://www.medcalc.org; 2019). Statistical analysis was performed with one-way ANOVA with Tukey’s multiple comparison test, as post-hoc test or Kruskall-Wallis and Dunn’s test, as appropriate. To assess normality of variance changes Kolmogorov-Shapiro normality test was used. Results are expressed as mean value ± standard error (SE). The value of p<0.05 was considered the criterion for statistical significance.

## Results

### OCA decreases gelatinase activity and enzyme release in the bile of rats submitted to I/R

**Fig 1:**
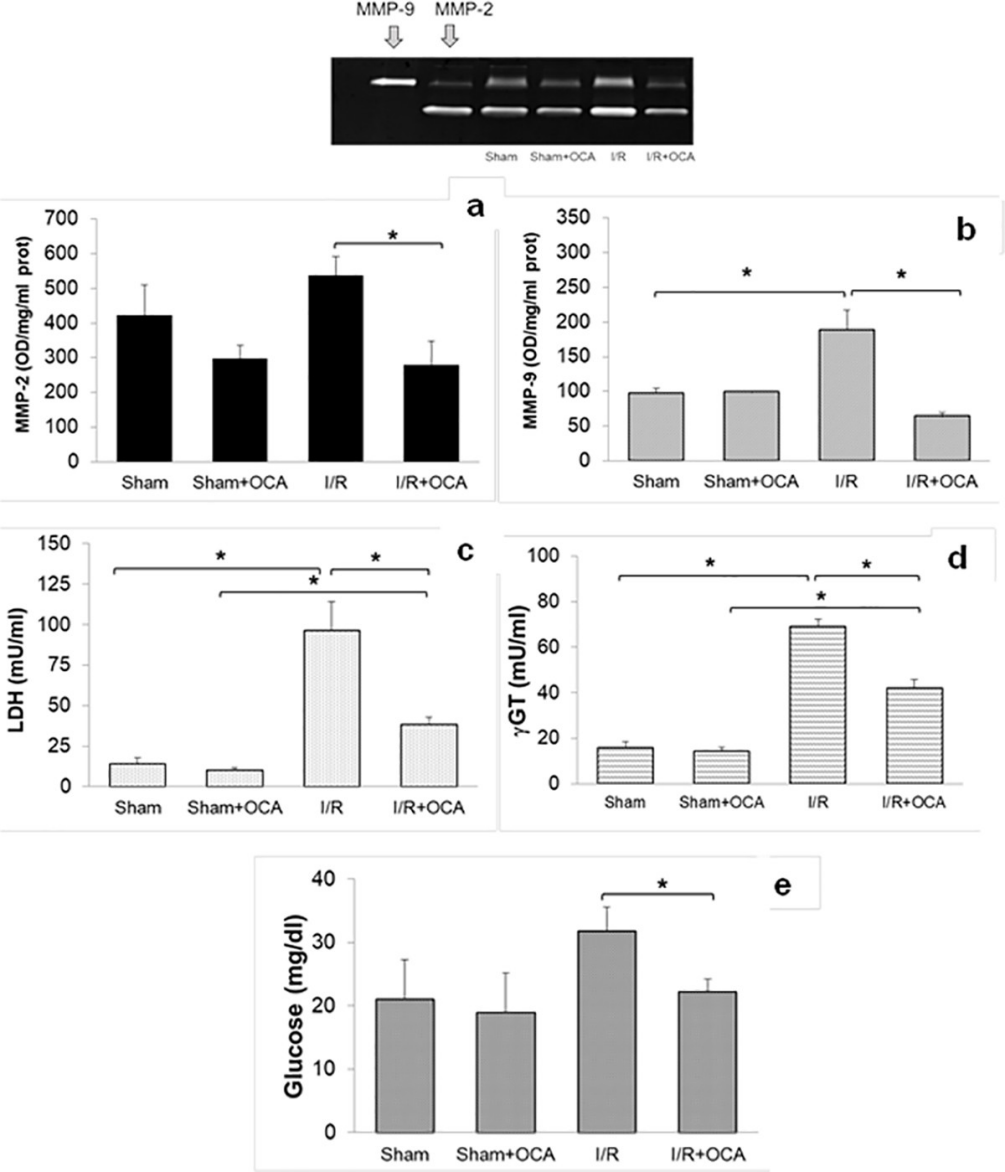
MMP-2 and MMP-9 activity, LDH, γGT and glucose levels were evaluated in bile at the end of reperfusion period. Livers were submitted to 60 min ischemia followed by 60 min reperfusion. Sham-operated control animals underwent similar manipulation without vascular occlusion. (a): p<0.05; (b): p<0.01; (c): p<0.01; (d) p<0.01; (e): p<0.05. The results are reported as the mean ±SE; total animals = 24, n = 6 each group. Matrix metalloproteinase-2 (MMP-2), matrix metalloproteinase-9 (MMP-9), lactate dehydrogenases (LDH), γ-Glutamyltransferase (γGT), obeticholic acid (OCA).

In I/R group treated with OCA a net decrease in MMP-2 and MMP-9 activity was observed in bile, compared to the vehicle-treated I/R group (Fig 1A and 1B). The same trend was detected in the release of enzymes into the bile, LDH and γGT as well as glucose: a significantly lower content of LDH, γGT and glucose in I/R rats treated with OCA was observed compared to the vehicle-treated I/R group (Fig 1C–1E). No difference in biliary content of MMP-2 and MMP-9 activity, LDH, γGT and glucose levels between sham-operated and sham-operated rats treated with OCA was found (Fig 1C–1E).

A correlation between biliary MMP-2 and MMP-9 activity versus enzyme release was evaluated using I/R groups. A positive correlation was found comparing biliary MMP-2 and MMP-9 activity versus vs LDH (Table 1). The same trend occurred for MMPs versus γGT (Table 1).

**Table 1:** Correlation between biliary levels of MMPs versus biliary enzymes, glucose and ADMA in the I/R groups.

	Biliary MMP-2		Biliary MMP-9	
	r/r_s_	*P*	r/r_s_	*P*
LDH	0.600	<0.05	0.775	<0.01
γGT	0.679	<0.05	0.714	<0.05
Glucose	0.518	Ns	0.441	ns
ADMA	-0.607	<0.05	-0.802	<0.01

Matrix metalloproteinase-2 (MMP-2), matrix metalloproteinase-9 (MMP-9). lactate dehydrogenases (LDH), γ-Glutamyltransferase (γGT), asymmetric dimethylarginine (ADMA).

ns: not significant.

OCA treatment increased biliary ADMA after I/R as compared with vehicle-treated animals (μmol/l: 0.49±0.04 versus 0.26±0.05, respectively, p = 0.01). The same trend occurred between the sham-operated and OCA-treated sham-operated groups (μmol/l: 0.074±0.02 versus 0.19±0.03, respectively, p = 0.01). The correlation between biliary ADMA and MMPs was quantified using I/R groups: an inverse correlation was found between MMP-9 and MMP-2 versus ADMA levels (Table 1).

No difference in bile flow was detectable comparing I/R versus I/R + OCA group (μl/min/gr liver: 0.83±0.21 versus 0.96±0.30, respectively) and the same occurred for Sham-operated versus Sham-operated + OCA group (μl/min/gr liver: 1.89±0.22 versus 2.01±0.26, respectively).

**Fig 2:**
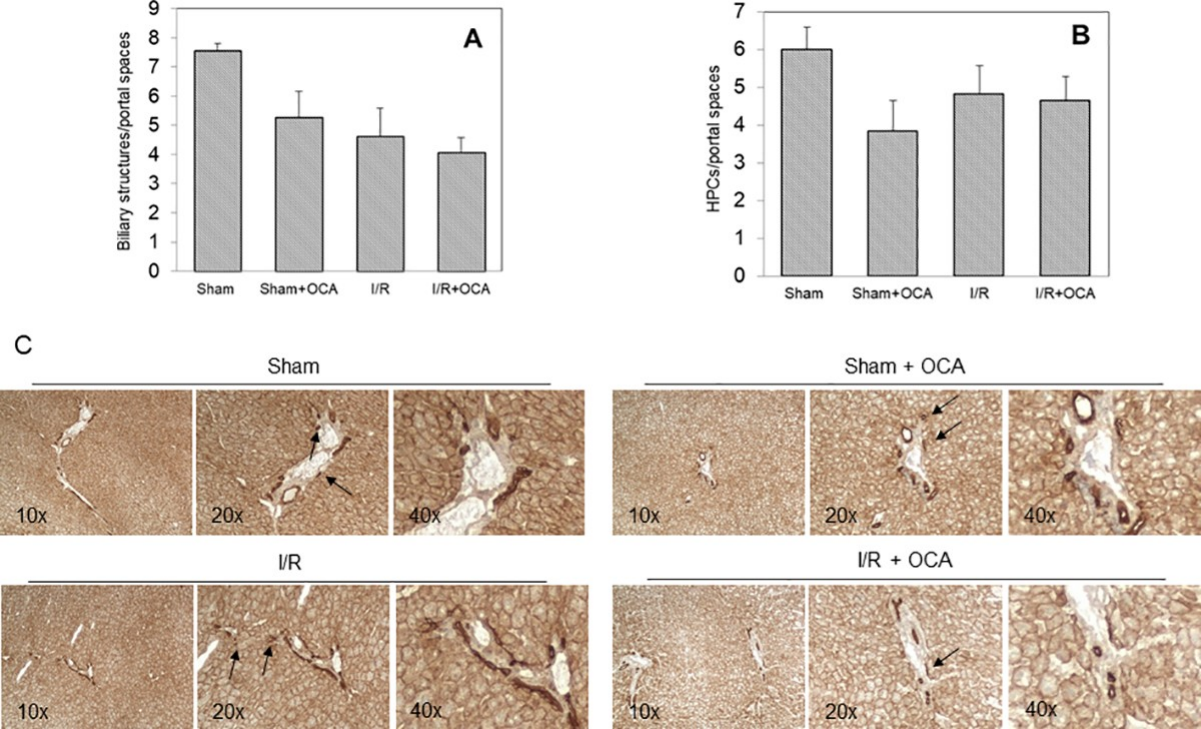
Amount of biliary structures and hepatic progenitor cells (HPCs) following I/R. Livers were submitted to 60 min ischemia followed by 60 min reperfusion. Sham-operated control animals underwent similar manipulation without vascular occlusion. Counts of biliary structures (a) and HPCs (b) were normalized on portal tracts. (c) representative micrographs of specimens from Sham-operated, Sham-operated + OCA, I/R and I/R + OCA rats showing the morphology of the liver. Biliary structures are outlined by a dark brown staining within or at the periphery of the portal tract, while HPCs (black arrows) are single cells or small clumps disperse at periphery or in the liver parenchyma. Magnification: (c) 10X and 20X. The results are reported as the mean ±SE; total animals = 24, n = 6 each group. Obeticholic acid (OCA).

To further characterize the cholangiocyte integrity, a morphometric analysis was performed to quantify biliary structures and hepatic progenitor cells (HPC). Biliary structures were manually counted, taking into account the expression of pCK and the phenotypic features of HPC and of reactive ducts deriving from the intrahepatic biliary tree, as described by Roskams et al. [21] and by us [22, 23]. Counts of biliary structures (Fig 2A and 2C) and HPCs (Fig 2B and 2C), normalized on portal tracts, reveal no differences between rats following I/R or sham-operated with and without concomitant OCA treatment.

**Fig 3:**
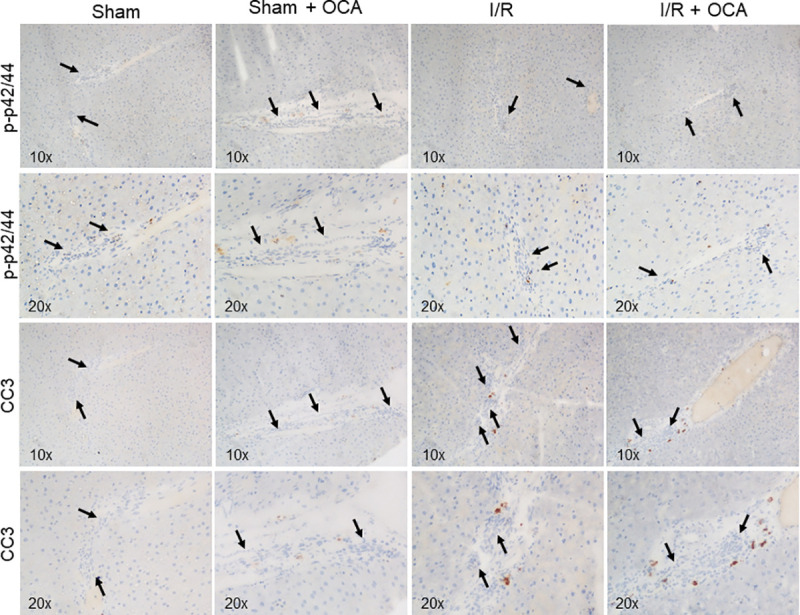
Cell proliferation (p-p42/44 MAPK) and apoptosis (cleaved Caspase-3) following I/R. Livers were submitted to a 60 min ischemia followed by 60 min reperfusion. Sham-operated control animals underwent similar manipulation without vascular occlusion. Arrows indicate the biliary structures in snap-frozen slides from rats undergone treatments with or without OCA challenges. cleaved Caspase-3 (CC3), obeticholic acid (OCA).

We performed immunostainings to evaluate cell proliferation (p-p42/44 MAPK) and apoptosis (cleaved Caspase-3); both proliferation and apoptosis markers were very faint and unevenly expressed (Fig 3). This is consistent with the immunohistochemical data showing only a very mild ductular proliferation following the different treatments in rats (Fig 2).

### OCA decreases hepatic gelatinase activity and increases RECK and TIMP protein expression in livers submitted to I/R

**Fig 4:**
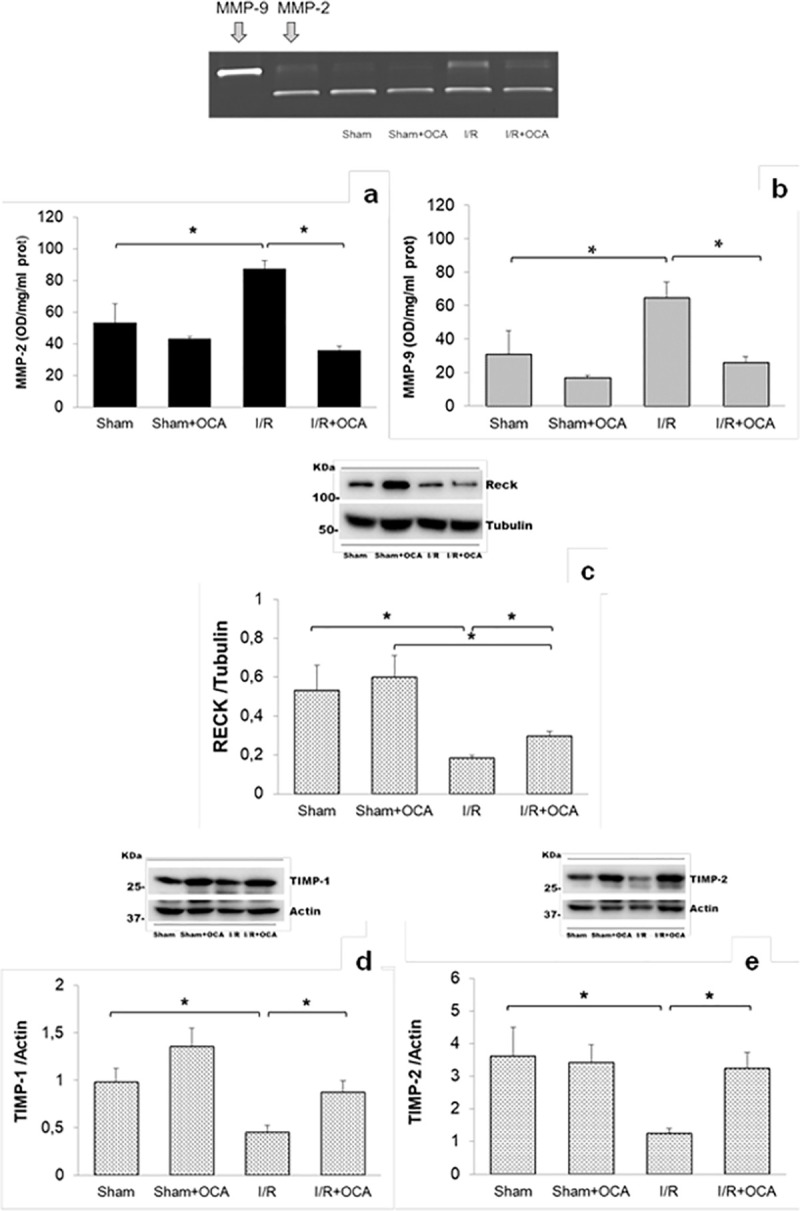
Hepatic MMP-2 and MMP-9 activity, RECK, TIMP-1 and TIMP-2 at the end of reperfusion. Livers were submitted to 60 min ischemia followed by 60 min reperfusion. Sham-operated control animals underwent similar manipulation without vascular occlusion. (a) p<0.01; (b) p<0.01; (c) p<0.01; (d) p<0.05; (e) p<0.01. The results are reported as the mean ±SE; total animals = 24, n = 6 each group. Matrix metalloproteinase-2 (MMP-2), matrix metalloproteinase-9 (MMP-9), reversion-inducing cysteine-rich protein with Kazal motifs (RECK), tissue inhibitor of metalloproteinase-1 (TIMP-1), tissue inhibitor of metalloproteinase-2 (TIMP-2), obeticholic acid (OCA).

Liver MMP-2 and MMP-9 activity increases after I/R when compared with sham-operated rats (Fig 4A and 4B). A significant decrease in hepatic MMP-2 and MMP-9 activities was detected in the OCA-treated I/R group compared with vehicle-treated I/R rats. No difference in MMPs activity emerged when compared sham-operated versus sham-operated treated with OCA (Fig 4).

The results of RECK protein analysis in the liver show marked decrease in the I/R group compared to the respective sham-operated group (Fig 4C). A significant upregulation of RECK was found in the I/R group treated with OCA compared to the vehicle-treated I/R group. Comparable RECK protein values were observed in both sham-operated and OCA-treated sham-operated groups. The same trend was found for TIMPs: an increase in liver TIMP-1 and TIMP-2 was detected in the I/R group treated with OCA compared to the vehicle-treated I/R group (Fig 4D and 4E). Comparable TIMP-1 and TIMP-2 protein values were found in both sham-operated and OCA-treated sham-operated groups.

**Fig 5:**
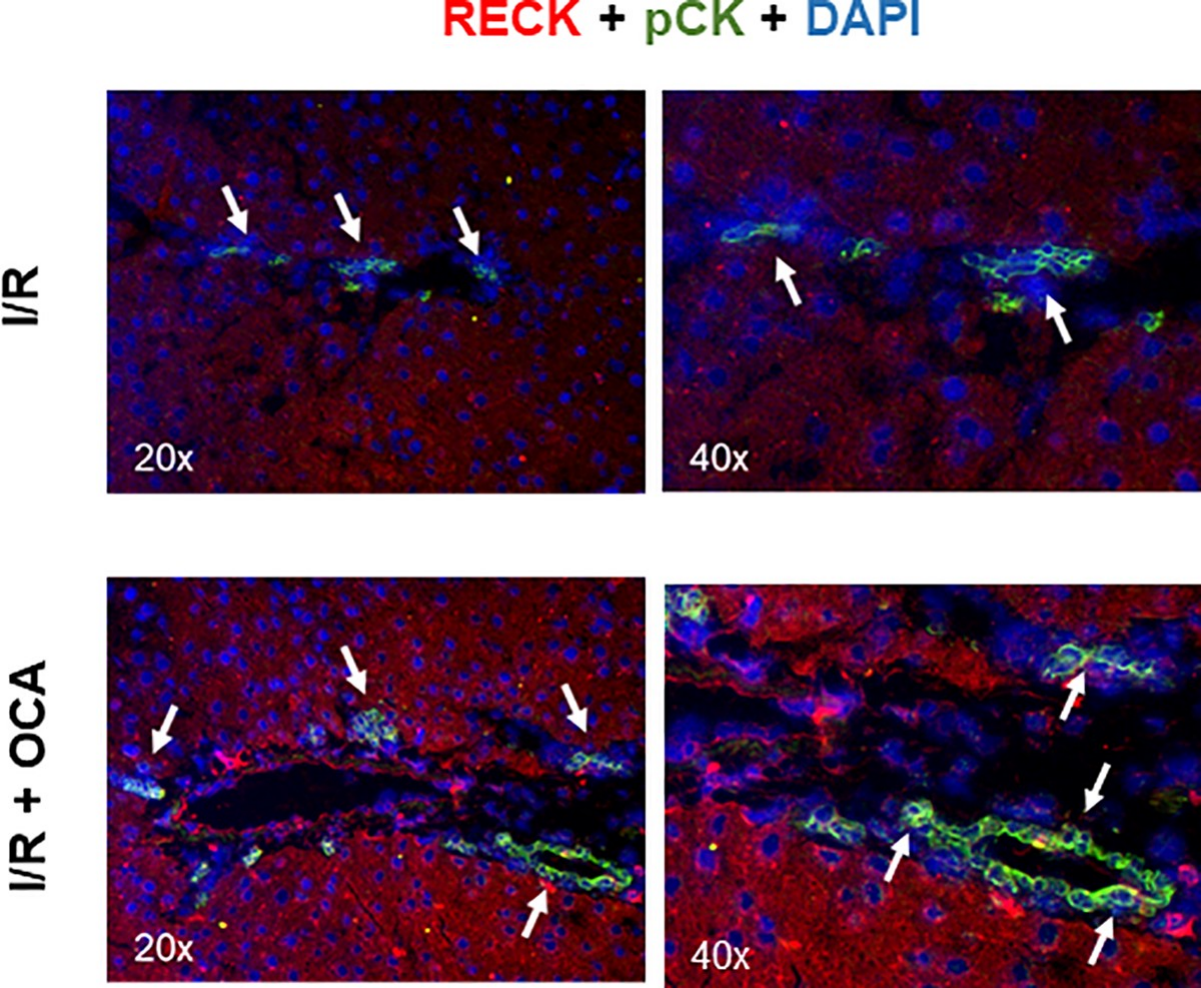
Hepatic RECK localization at the end of reperfusion. Livers were submitted to 60 min ischemia followed by 60 min reperfusion in rats treated with or without OCA. Cytokeratin (pCK) was used as marker of biliary structures. Reversion-inducing cysteine-rich protein with Kazal motifs (RECK), obeticholic acid (OCA).

To evaluate the source of RECK, we performed immunostainings of hepatic tissue demonstrating that RECK expression is mainly localized in both cholangiocytes and hepatocytes (arrows) in I/R group treated with OCA as reported in Fig 5 and S1 Fig.

The correlation between tissue MMP activity versus RECK was evaluated in I/R groups. An inverse correlation between MMP-2 and MMP-9 versus RECK was found (Table 2). Furthermore, an inverse correlation between MMP-2 and MMP-9 versus TIMPs was found (Table 2).

**Table 2:** Correlation between hepatic levels of MMP activity versus liver RECK, TIMPs and iNOS in the I/R groups.

	Hepatic MMP-2		Hepatic MMP-9	
	r/r_s_	*P*	r/r_s_	*P*
RECK	-0.791	<0.05	-0.716	<0.05
TIMP-1	-	-	-0.828	<0.05
TIMP-2	-0.927	<0.01	-	-
iNOS	0.831	<0.01	0.666	<0.05

Matrix metalloproteinase-2 (MMP-2), matrix metalloproteinase-9 (MMP-9), reversion-inducing cysteine-rich protein with Kazal motifs (RECK), tissue inhibitor of metalloproteinase-1 (TIMP-1), tissue inhibitor of metalloproteinase-2 (TIMP-2), inducible nitric oxide synthase (iNOS).

OCA treatment decreased iNOS expression after I/R as compared with vehicle-treated animals (iNOS/tubulin: 1.57±0.18 versus 1.03±0.17, respectively, p<0.05). No significant changes emerged when comparing sham-operated and OCA-treated sham-operated groups (iNOS/tubulin: 0.93±0.14 versus 0.96±0.15, respectively). A positive correlation between MMP-2 and MMP-9 vs. iNOS was observed (Table 2).

### OCA reduces serum gelatinase activity in rats submitted to hepatic I/R

A decrease in MMP-2 and MMP-9 activity was found in the serum of I/R rats treated with OCA compared with vehicle-I/R treated rats (Table 3). MMP-9 activity increased in I/R rat serum compared with vehicle treated rats (Table 3). OCA administration induced a non-significant reduction of hepatic serum enzyme levels in the I/R group (Table 3).

**Table 3:** Effects of OCA on serum biochemical parameters and MMP activity in sham-operated and I/R rats.

	Sham-operated	Sham-operated + OCA	I/R	I/R + OCA
AST (mU/ml)	233±49	149±29	3622±899	2508±169
ALT (mU/ml)	68±15	49±9	3830±961	2810±223
ALP (mU/ml)	414±45	372±31	589±48	627±57
MMP-2 (OD/mg/ml prot)	72.2±5.1	72.2±8.4	82.9±2.5	60.3±3.0*
MMP-9 (OD/mg/ml prot)	79.3±6.1	80.5±2.6	104.2±6.3#	65.9±6.0**

Obethicolic acid (OCA), aspartate transaminase (AST), alanine transaminase (ALT), alkaline phosphatase (ALP), matrix metalloproteinase-2 (MMP-2), matrix metalloproteinase-9 (MMP-9).

*P<0.05 vs I/R

**P<0.01 vs I/R and

#*P* <0.01 vs Sham-operated.

## Discussion

This work, for the first time, shows that the FXR agonist OCA decreases MMP-2 and MMP-9 activity in tissue and bile obtained from livers submitted to I/R injury. This is associated with the ability of OCA to restore the RECK level decreased by ischemic insult. The decreased levels of MMPs in bile occurring in the OCA-treated group during hepatic I/R injury are inversely correlated with biliary ADMA, but positively correlated with biliary enzymes.

FXR plays a pivotal role in physiopathology of the liver and the biliary tree. Initially identified as a regulator of bile acid homeostasis, known FXR functions now range from control of cholesterol, lipid and glucose metabolism to its role in inflammation, liver regeneration and carcinogenesis [24]. The beneficial effects of OCA have been related to its anti-cholestatic, anti-inflammatory and anti-fibrotic properties [25] and recently the therapeutic value of OCA has been documented in cholangiocarcinoma progression by regulating cell proliferation, migration and mitochondrial energy metabolism [26].

Our study provides novel insights into the effects of OCA on the control of MMP-2 and MMP-9 activity in bile. Using samples obtained from the sham-operated group, we have, for the first time, documented high content in MMPs in bile: up to 8-fold for MMP-2 and up to 3-fold for MMP-9 when compared with their levels in liver tissue. In addition, we have demonstrated a novel role for OCA in the modulation of biliary MMP activity: MMP-2 and MMP-9 recovery was observed in the I/R group treated with OCA. Moreover, a similar trend occurred for markers of cholangiocyte injury (LDH and γGT) and cholangiocyte function (glucose) [27] in the I/R group treated with OCA. These events are associated with the ability of OCA to increase biliary ADMA via MATE-1 upregulation, the latter a canalicular efflux transporter involved in ADMA clearance [6]. Although no published data on biliary content in MMPs and ADMA have been reported, the correlation between biliary MMPs and cholangiocyte injury and the correlation between biliary ADMA and MMPs suggest that both MMPs and ADMA levels could play a crucial role in the modulation of cholangiocyte integrity during I/R damage. Despite these results, the morphometric quantification of biliary structures and HPCs reveal comparable features between rats following I/R with and without concomitant OCA treatment. HPCs, previously known as oval cells, were first described in rats following bile duct ligation [28]. HPCs, in normal conditions, reside in the canals of Hering in an inactive state, but, following liver insults of different origin, this cell population can proliferate to restore the normal hepatic homeostasis. HPCs have an oval shape, with scant cytoplasm and big nucleus, and can be found isolated or in small clumps at the periphery of the portal tract or into the liver parenchima [21]. This cell population is characterized by the coexistence of markers typical of different cell types, such as Keratin 7 and 19 (biliary markers), Keratin 18 (hepatocyte marker), NCAM and chromogranin (neuroendocrine compartment) [29, 30].

We hypothesized that the development of histological alteration could be observed increasing the reperfusion period while changes in biliary LDH levels represent an early detectable event. In addition, a recent clinical study documented that a biliary LDH concentration less than 3700 U/L, as also found in our study, has been associated with low histological intrahepatic bile duct injury [31].

Bile plays an important role in digestive physiology, especially in the emulsification and digestion of fat. Packialakshmi et al. have suggested that biliary MMPs take part in the digestion of collagen proteins [32]. It is very likely that in bile MMPs play other gastrointestinal roles, such as activating growth factors, antimicrobial proteins, and other proteases, including MMPs, and several receptor proteins, which still need to be explored [33, 34].

Many studies suggest that MMPs are inversely regulated by RECK, a membrane-anchored glycoprotein key regulator of ECM integrity and angiogenesis. RECK is linked to the cell membrane and can inhibit the activity of MMP-2, MMP-9 and MMP-14 through different mechanisms, such as direct inhibition of protease activity, regulation of their release from the cell and sequestration of MMPs at the cell surface [35–38]. In mice fatty livers RECK has been identified as a novel transcriptional target gene of the FXR receptor [9]. Moreover, the administration of an FXR agonist directly augmented hepatic RECK expression [9]. Since RECK regulates the transcription and secretion of MMP-9 and directly inhibits its enzymatic activity [28, 31], we hypothesized that OCA may suppress the hepatic activity of MMP-2 and MMP-9 by promoting the liver expression of RECK during hepatic I/R injury. Previous data have documented the capacity of OCA to modulate MMP-2 expression in hepatic stellate cells promoting resolution of liver fibrosis [39].

Several studies demonstrated that MMPs and TIMPs have important roles in the preservation of liver homeostasis [40]. In particular, TIMP-1, the major endogenous regulator of MMP-9, plays a protective function in the control of survival and proliferation of liver cells during I/R injury [41]. As recently reported, TIMP-1 and TIMP-2 have been found significantly decreased at different stages of hepatic I/R damage [42] and our data support these results. We further demonstrated the OCA’s ability in the recovery of hepatic TIMP expression to the levels comparable to those observed in the sham-operated rats. Several experimental models of liver I/R injury have demonstrated protective effects of MMP inhibitors vis-à-vis cell necrosis, apoptosis and rearrangement of the ECM [43]. Hepatic I/R is correlated with MMP gene expression, activation and release, with a significant effect on tissue integrity [44]. Gelatinases play an important role as they are the main components of Disse's space [45]. Increased activity of these MMPs may cause liver injury, with alterations of the sinusoidal cells and remodeling of the stromal structure.

Extensive evidence supports a role for non-parenchymal cells, cholangiocytes, sinusoidal endothelial cell (SECs), hepatic stellate cells (HSCs), Kupffer cells (KCs) and other immune cells, including lymphocytes, in the production of MMPs and their specific inhibitors TIMPs [46]. Several studies have shown that MMP2, one of the most studied enzymes in liver fibrosis, is mainly secreted by KCs and by HSCs while MMP-9 (gelatinase B) is predominantly expressed in KCs but can be also expressed by neutrophils, macrophages and fibroblasts [43].

NO can be generated from three isoforms: neuronal NOS (nNOS or NOS1), inducible NOS (iNOS or NOS2), and endothelial NOS (eNOS or NOS3). Under normal conditions, only eNOS is present in the liver; an excess production of NO, generated primarily by iNOS as a triggering mechanism, has been implicated as a mediator of cellular injury at sites of inflammation, including hepatic I/R injury [47]. Under these inflammatory conditions, a concurrent upregulation of both iNOS and MMP-9 could take place [48]. Recent studies have suggested that iNOS expression has a regulatory function on MMP-9 activation in liver I/R injury [14]: iNOS inhibition markedly down-regulates MMP-9 expression and activity. RECK regulates MMP-9 through the suppression of its secretion from cells and through direct enzymatic activity. Our data show a RECK increase, after OCA treatment, concomitant with a significant decrease in MMP activity and iNOS content in livers after I/R injury. Since RECK can regulate ECM remodeling, we can thus posit a regulatory role for OCA in the modulation of hepatic injury mediated by MMPs.

Results obtained using a rat model of intestinal I/R injury have also demonstrated that pretreatment with OCA preserves mucosal integrity, inhibits bacterial translocation and reduces pro-inflammatory cytokine release [49].

The limit of this study is the lack of analysis of intrahepatic and extrahepatic ducts. A recent study of Aloia et al. reported as little is known about what happen to cholangiocytes during I/R injury [50]. Recent findings has been shown existence of an adaptation mechanisms of cholangiocytes to the damage [51]. The expression and intracellular distribution of MMP in cholangiocytes of intrahepatic and extrahepatic ducts in a model of rat I/R injury would be useful in order to provide new therapeutic options for biliary diseases and it will be taken into account for future studies.

## Conclusion

**Fig 6:**
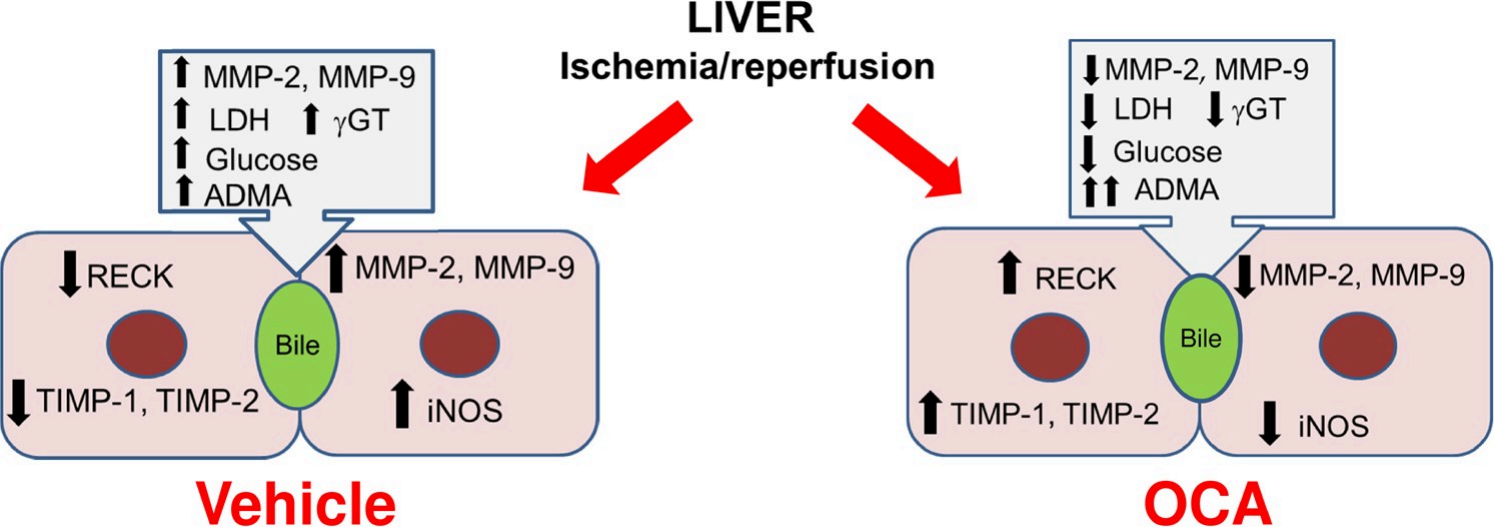
Schematic representation of changes in ADMA, iNOS and MMP pathway that occur in rat livers after I/R (vehicle) or I/R and OCA administration. Asymmetric dimethylarginine (ADMA), γ-Glutamyltransferaseγ (GT), inducible nitric oxide synthase (iNOS), lactate dehydrogenases (LDH), matrix metalloproteinase-2 (MMP-2), matrix metalloproteinase-9 (MMP-9), obeticholic acid (OCA), reversion-inducing cysteine-rich protein with Kazal motifs (RECK), tissue inhibitor of metalloproteinase-1 (TIMP-1), tissue inhibitor of metalloproteinase-2 (TIMP-2).

Our results demonstrate the ability of OCA to limit the hepatic activation of MMP-2 and MMP-9 activity that occurs during hepatic I/R damage, probably via timely recovery of the RECK and TIMP proteins. In addition, high levels of biliary MMPs were for the first time documented, as well as their modulation by OCA treatment (Fig 6). Although the role of biliary MMPs is still unclear, this study shows an inverse correlation with ADMA. OCA administration increases biliary ADMA and protects cholangiocytes via downregulation of biliary MMPs. This event is supported by a positive correlation between MMP activity versus biliary enzymes, an index of cholangiocyte injury. OCA thus appears to confer protection against cholangiocyte damage in livers submitted to I/R.

The present study demonstrates that OCA is involved in the control of the ADMA/iNOS/MMP pathway in livers submitted to I/R injury.

## Supporting information

S1 Fig (TIF)

S1 Raw images (PDF)
