# Supplementary figures and images for: Obeticholic acid reduces biliary and hepatic matrix metalloproteinases activity in rat hepatic ischemia/reperfusion injury
Source: PLoS One. 2020 Sep 10;15(9):e0238543. doi: 10.1371/journal.pone.0238543 (PMC7482919; doi:10.1371/journal.pone.0238543)

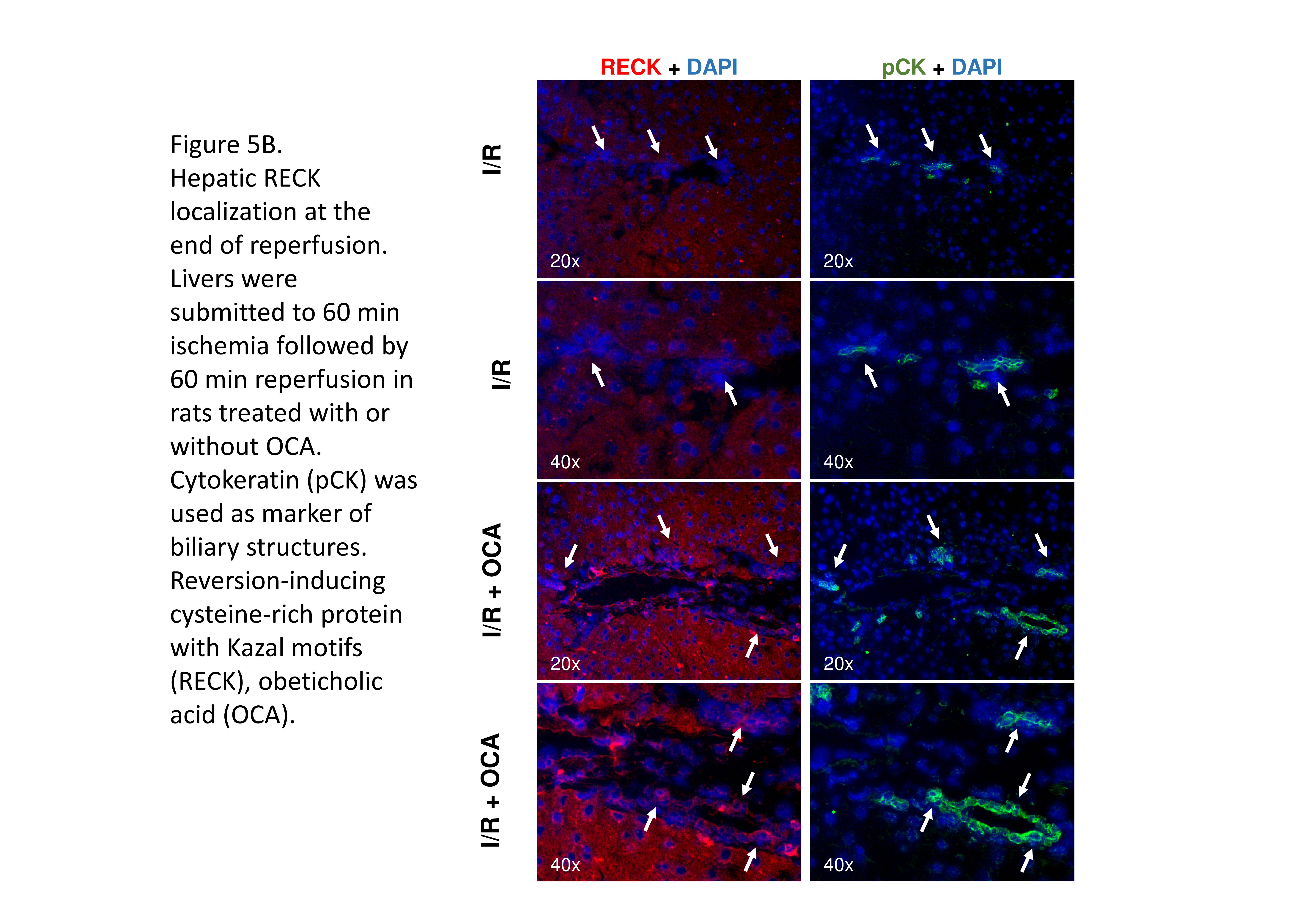

Supplement: S1 Fig — (TIF) [file pone.0238543.s001.tif]
