# Supplementary material for: Obeticholic acid reduces biliary and hepatic matrix metalloproteinases activity in rat hepatic ischemia/reperfusion injury
Source: PLoS One. 2020 Sep 10;15(9):e0238543. doi: 10.1371/journal.pone.0238543 (PMC7482919; doi:10.1371/journal.pone.0238543)
Supplement: S1 Raw images — (PDF) [file pone.0238543.s002.pdf]

RAW DATA OF
WESTERN BLOT ANALYSIS

RECK (106 KDa) GEL A and B

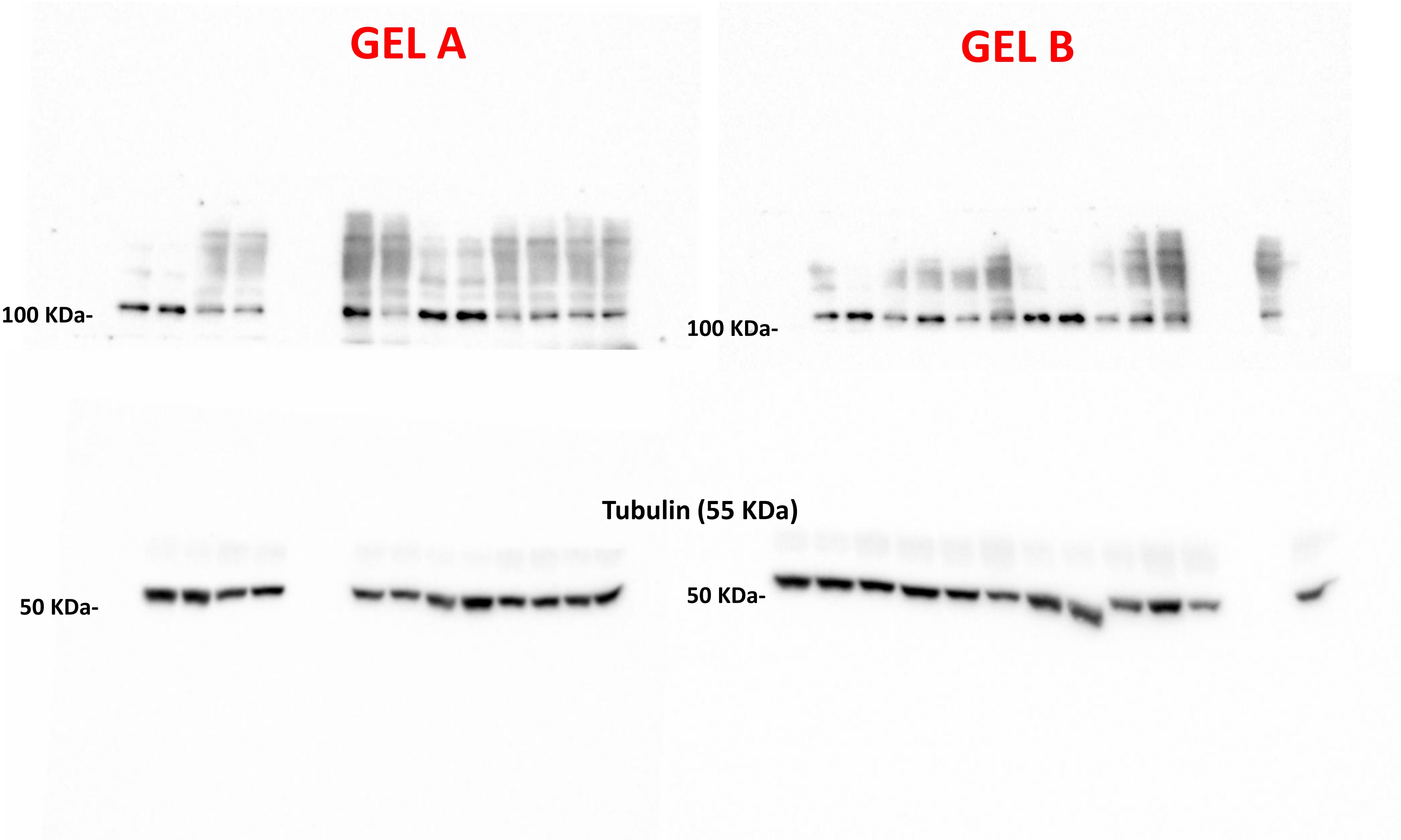

Precision Plus molecular weights all blue (Bio-Rad Laboratories) were used.

RECK (106 KDa) Gel C

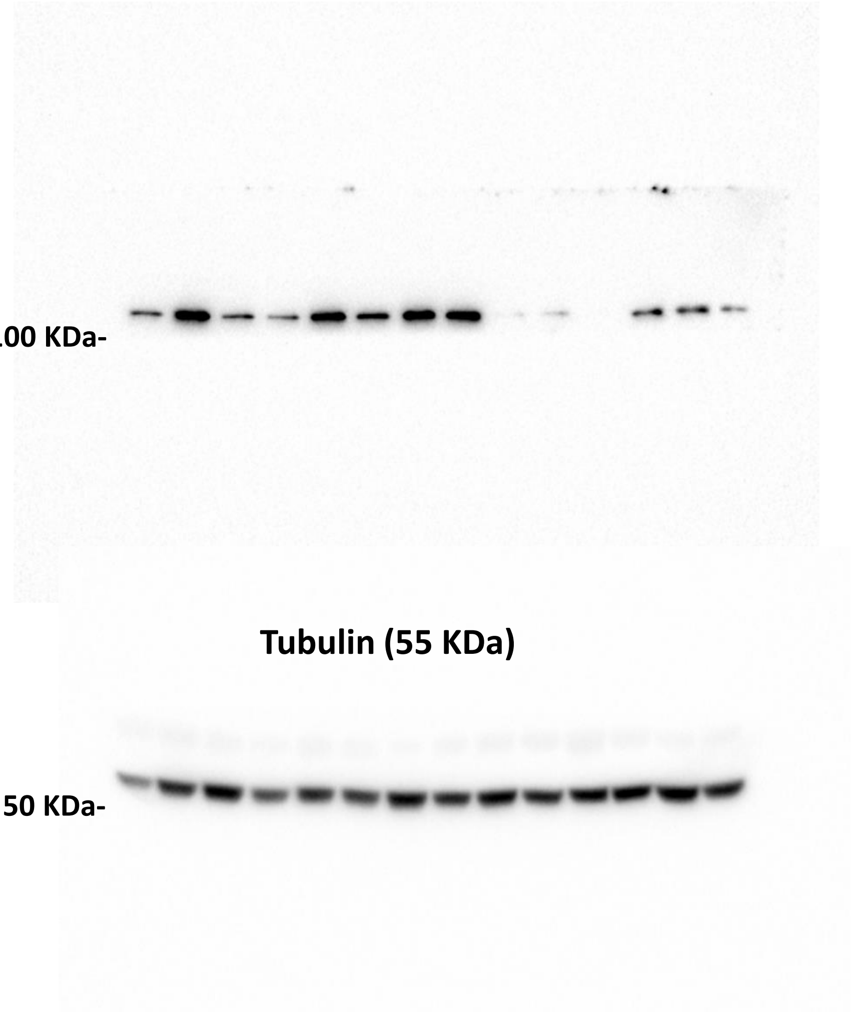

Precision Plus molecular weights all blue (Bio-Rad Laboratories) were used.

RECK: LIST OF SAMPLES

Gel A	Gel B	Gel C
1 Sham	1 Sham	1 Sham
2 Sham + OCA	2 Sham + OCA	2 Sham + OCA
3 I/R	3 I/R	3 I/R
4 I/R + OCA	4 I/R + OCA	4 I/R + OCA
5 ----	5 I/R 120	5 I/R 120
6 ----	6 I/R 120 +OCA	6 I/R 120 +OCA
7 I/R 120	7 Sham	7 Sham
8 I/R 120 + OCA	8 Sham + OCA	8 Sham + OCA
9 Sham	9 I/R	9 I/R
10 Sham + OCA	10 I/R + OCA	10 I/R + OCA
11 I/R	11 I/R + OCA	11 I/R 120
12 I/R 120	12 ----	12 I/R 120 +OCA
13 I/R 120	13 ----	13 I/R
14 I/R 120 + OCA	14 I/R 120+ OCA	14 I/R

TIMP-1 (28 KDa) GEL A and B

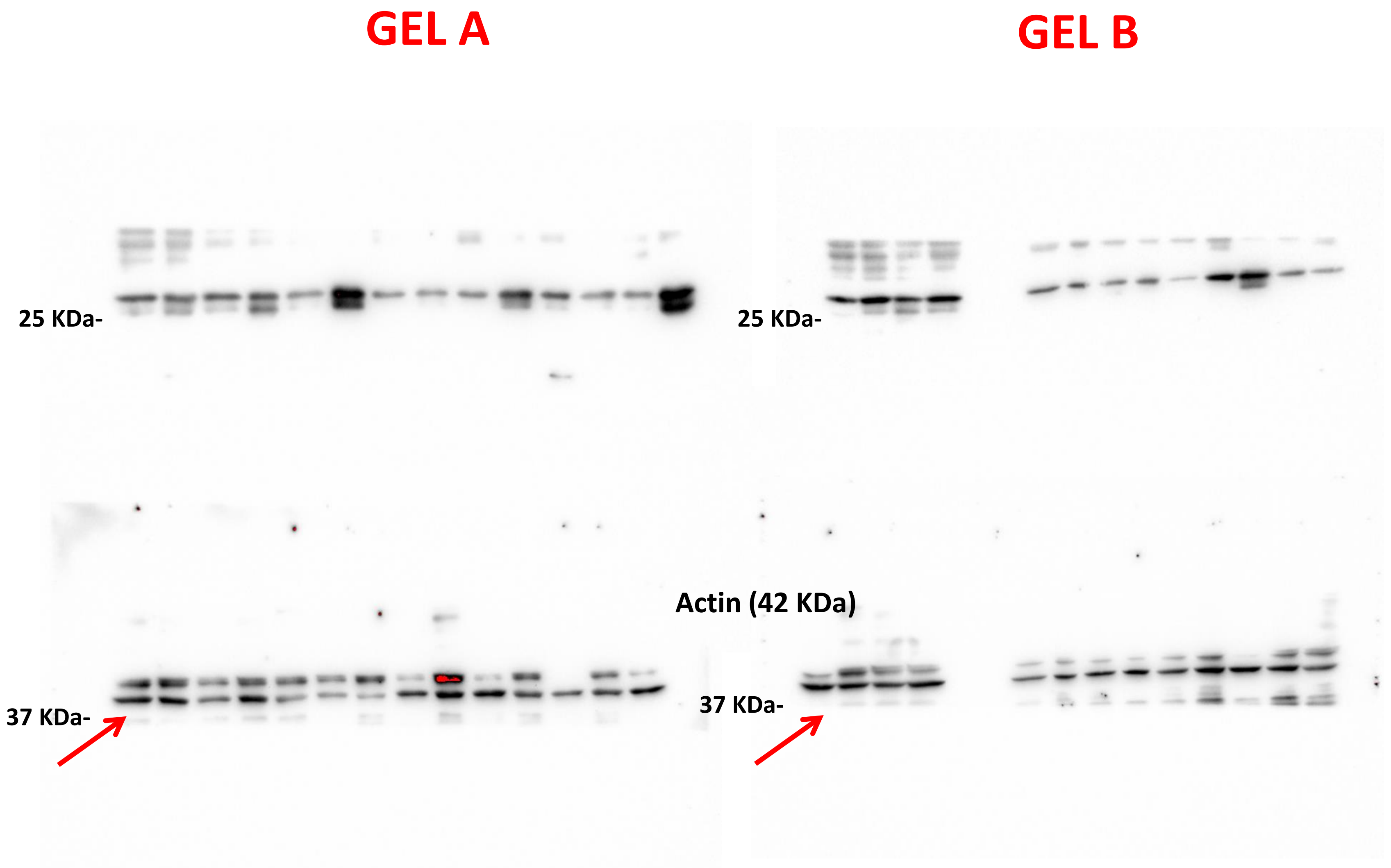

Precision Plus molecular weights all blue (Bio-Rad Laboratories) were used.

TIMP-1: LIST OF SAMPLES

Gel A	Gel B
**1 I/R**	**1 Sham**
**2 I/R**	**2 Sham + OCA**
**3 I/R 120**	**3 I/R + OCA**
**4 I/R**	**4 I/R + OCA**
**5 Sham**	**5------**
**6 Sham 120**	**6 Sham**
**7 Sham + OCA**	**7 Sham + OCA**
**8 I/R + OCA**	**8 I/R**
**9 Sham + OCA**	**9 I/R + OCA**
**10 I/R + OCA**	**10 I/R 120**
**11 Sham + OCA**	**11 Sham 120**
**12 I/R + OCA**	**12 I/R**
**13 Sham**	**13 I/R + OCA**
**14 I/R**	**14 Sham**

TIMP-2 (24.4 KDa) GEL A and B

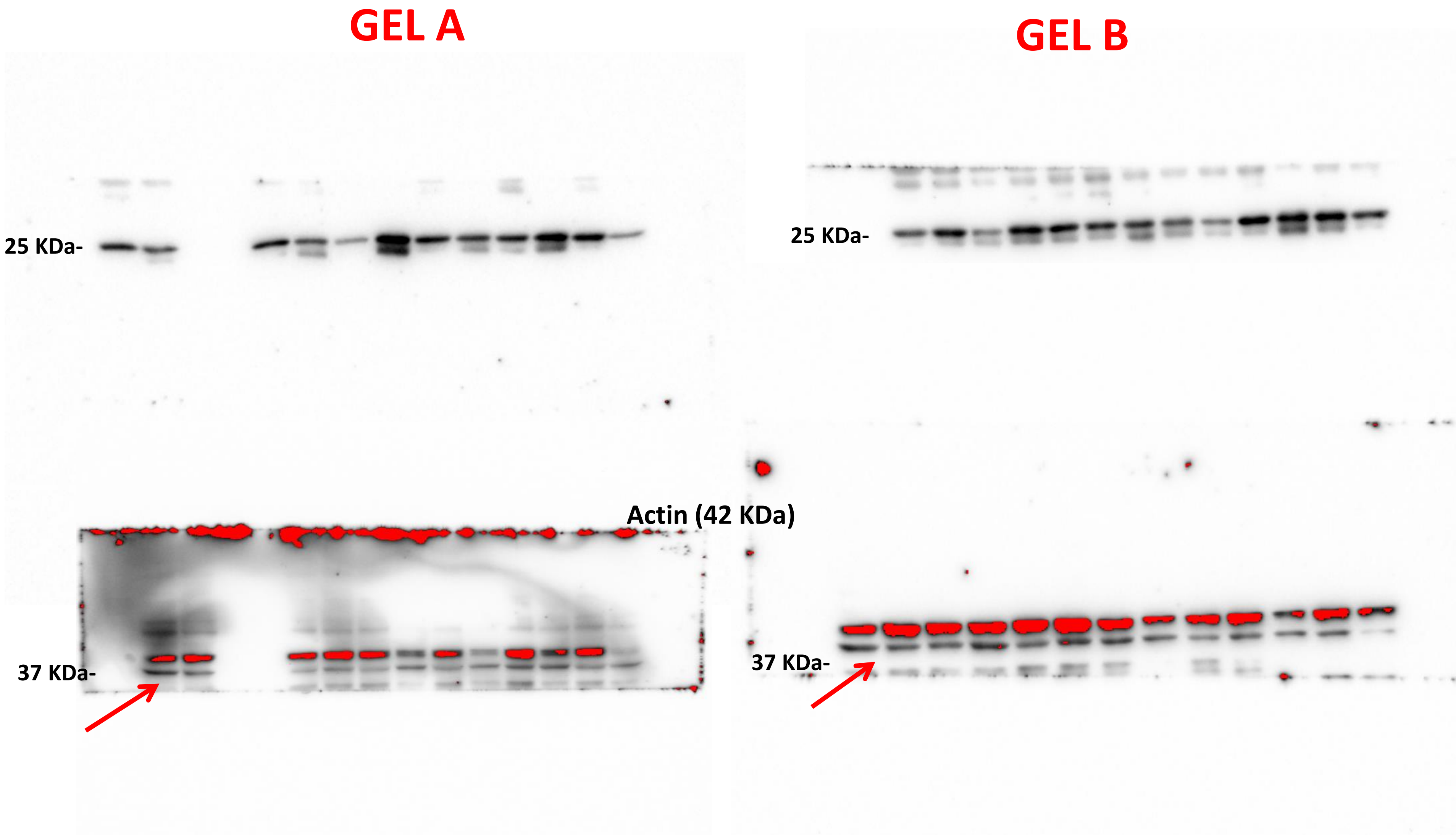

Precision Plus molecular weights all blue (Bio-Rad Laboratories) were used.

TIMP-2: LIST OF SAMPLES

Gel A

1 I/R + OCA
2 I/R

3 I/R + OCA out
4 I/R
5 I/R + OCA
6 I/R
7 Sham + OCA
8 Sham 120
9 Sham
10 Sham 120
11 Sham
12 I/R 120

Gel B

1 Sham
2 Sham + OCA
3 I/R
4 I/R + OCA
5 Sham
6 Sham + OCA
7 I/R
8 I/R + OCA
9 Sham
10 Sham + OCA
11 Sham + OCA
12 I/R + OCA
13 Sham
